# Art of Infant Feeding

**Published:** 1888-10

**Authors:** W. I. Thayer


### ART OF INFANT FEEDING.

BY W. I. THAYER, D. D. S., M. D.

The large mortality amongst infants is largely due to improper feeding, especially in summer. How best to feed infants, so as to nutrify *every* tissue that none may lack, has been the subject of much controversy.

Some hold that there is no good substitute for mother's milk, and all are pretty well agreed that human milk for infantile nourishment under proper conditions is quite near the sum of perfection. There is, however, a large majority of mothers who are, from good and sufficient reasons, incapable of properly nourishing their young; and, for reasons noted below, the number of good wet nurses is quite as limited.

It is quite probable that the reader does consider it important that *every* tissue of the infant *should be properly* and *fully nourished.* But there are a large number of children being fed naturally and artificially that are *not fully nourished.* It is not to be understood by this, that the adipose, epidermoid, cellular, elastic or fibrous tissues are not pretty well provided for, even in quite indifferent feeding, but the interests of the petrous tissues have seldom been considered, and the painful exhibit is patent everywhere by edentulous persons, and mouths full of badly broken-down and rapidly decaying teeth. The reason of this is because the teeth have not been furnished with the lime salts in due proportion to their demands. Since it is the calcareous matter that enables the teeth to resist most all disintegrating influences, and since they have been deprived of this pabulum, we find effect to follow cause here as well as in every other condition.

In the late American Medical Association, held May 9, 1888, it was recommended to feed, artificially, infants on barley and rice water with milk or cream and lime water. One cannot put calcareous matter into teeth by lime—carbonate of lime—water, because there is ten times more of the *phosphate* of lime in tooth composition than of the carbonate. A child can, by no possible means, get any of the inorganic constituents out of either rice or barley water, and but a poverty of supply out of cream. Calcareous matter cannot be gotten out of a mammary gland or out of a bottle if it has not been first put there.

Good fresh cow's milk is a good substitute for human milk, if, and provided, some method will be used to partly predigest the tough casein found therein. Children, as a whole, cannot digest such casein. If the nurse will partly digest the milk before ingestion, then all this difficulty will be overcome. But how many mothers are capable of doing it? If too much time is given, the fluid will be made bitter; if too much heat is applied, the digestive ferment is ruined and no digestion has taken place. By the most reliable analysis of human milk that we are able to find vemois, bacquerel and meigs, from over forty-five different women, we find the casein to be 1.046, while in a fair average of cow's milk, it runs up as high as 3.022 per cent. Then thoroughly desiccated casein of cow's milk cannot again be dissolved, and is insoluble in strong caustic soda. Hence, the great necessity of predigesting this form of infant food.

All of these difficulties can be overcome in an artificial food, where the manufacturer will make provision *for the petrous tissues*, furnish a liberal supply of the albuminoids and partly pre-digest the different constituents of the artificial food, it is possible to attain unto an exceedingly valuable food for infants, in which *every tissue* can be properly nutrified, and pabulum can be furnished that will *digest as readily* as human milk. To elucidate our subject a little further, we will give the composition of some of the artificial foods now on the market, and leave the reader to judge of the soundness of our argument in providing for all of the tissues. * * * * * * * *

What is necessary in an artificial food for infants is, first, *ease of digestion*; second, a *high* percentage of the *albuminoids*; and lastly, but far from being the least, a *liberal supply of the inorganic constituents*, especially material to furnish the *phosphate* of lime for the *petrous tissues.* Even good muscular tissue requires some twenty per cent. of the inorganic constituents for their best composition.

The woman, as soon as she is aware that she is pregnant, should partake liberally of the coarser bread foods at least three times a day. Bread foods that are constructed out of the *meal* product of the grain partaken of, such as oat *meal*, graham bread, brown bread made of Indian *meal* and rye *meal*. *Bolted* wheat flour, cooked into any form of food, should *not* be eaten.

All of our grains in their first immediate environment, their husks, are rich in the various lime salts that are necessary to make stong teeth and bones. There is no other form of food that can be found that will furnish in a full and rightly balanced proportion, and so easy of division and appropriation as can be found in the whole of our wheat, oat, rye and corn.

The teeth begin to form as early as the sixth week of conception, and it is *when they are forming*, that they require to be fed with calcareous matter. Teeth once built up, *are built up forever.* After eruption, there is no repairing of dental cavies, except by the dentist. Therefore, it follows, that what calcareous matter passes through the umbilical cord, mammary glands and the bottle, is of the highest importance. Suppose that in human milk or infant foods, none of the proteius or albuminoids were to be found, where would the majority of the tissues be nourished? The same is true if the petrous tissues should be starved.

The starches cannot well be digested by infants under one year of age. These hydro-carbons are disposed of by the amyolytic ferments found in the saliva, pancreatic and intestinal juices. These ferments such children do not secrete in sufficient quantity to digest the starches.

In the natural process of competent digestion, the amyolytic ferments convert starch into *dextrine*, then into soluble sugar which can be immediately absorbed. The manufacturer of an artificial food for infants must immitate nature and convert his raw starches into dextrine by long baking at a temperature of some 350 degrees Fahrenheit. Then, again, his nitrogenous matter—cow's milk—he must partly pre-digest with prancreatine, and this latter, and even the first, process he can do more evenly and successfully, because he employs professional ability in an experienced chemist.

After the child begins to eat, it should have no food constructed out of the *bolted* wheat or grain used. He should continue to eat liberally of such course bread foods till the last permanent tooth has been erupted more than a year. In fine, if the suggestions of this paper are followed out, it will insure a good set of permanent teeth, that will be able to resist influences that now so easily melt and break them down.

